# Surfactant Protein-A Suppresses Eosinophil-Mediated Killing of *Mycoplasma pneumoniae* in Allergic Lungs

**DOI:** 10.1371/journal.pone.0032436

**Published:** 2012-02-23

**Authors:** Julie G. Ledford, Sambuddho Mukherjee, Michele M. Kislan, Julia L. Nugent, John W. Hollingsworth, Jo Rae Wright

**Affiliations:** 1 Department of Cell Biology, Duke University Medical Center, Durham, North Carolina, United States of America; 2 Department of Pulmonary, Allergy and Critical Care Medicine, Duke University Medical Center, Durham, North Carolina, United States of America; 3 Department of Immunology, Duke University Medical Center, Durham, North Carolina, United States of America; Louisiana State University, United States of America

## Abstract

Surfactant protein-A (SP-A) has well-established functions in reducing bacterial and viral infections but its role in chronic lung diseases such as asthma is unclear. *Mycoplasma pneumoniae* (Mp) frequently colonizes the airways of chronic asthmatics and is thought to contribute to exacerbations of asthma. Our lab has previously reported that during Mp infection of non-allergic airways, SP-A aides in maintaining airway homeostasis by inhibiting an overzealous TNF-alpha mediated response and, in allergic mice, SP-A regulates eosinophilic infiltration and inflammation of the airway. In the current study, we used an *in vivo* model with wild type (WT) and SP-A^−/−^ allergic mice challenged with the model antigen ovalbumin (Ova) that were concurrently infected with Mp (Ova+Mp) to test the hypothesis that SP-A ameliorates Mp-induced stimulation of eosinophils. Thus, SP-A could protect allergic airways from injury due to release of eosinophil inflammatory products. SP-A deficient mice exhibit significant increases in inflammatory cells, mucus production and lung damage during concurrent allergic airway disease and infection (Ova+Mp) as compared to the WT mice of the same treatment group. In contrast, SP-A deficient mice have significantly decreased Mp burden compared to WT mice. The eosinophil specific factor, eosinophil peroxidase (EPO), which has been implicated in pathogen killing and also in epithelial dysfunction due to oxidative damage of resident lung proteins, is enhanced in samples from allergic/infected SP-A^−/−^ mice as compared to WT mice. *In vitro* experiments using purified eosinophils and human SP-A suggest that SP-A limits the release of EPO from Mp-stimulated eosinophils thereby reducing their killing capacity. These findings are the first to demonstrate that although SP-A interferes with eosinophil-mediated biologic clearance of Mp by mediating the interaction of Mp with eosinophils, SP-A simultaneously benefits the airway by limiting inflammation and damage.

## Introduction

Eosinophils, the highly granular proinflammatory leukocytes important in the body's host defense against parasites, such as helminths, have also been implicated in the pathogenesis of many inflammatory conditions ranging from skin and gastroenteric diseases to allergy and bronchial asthma (reviewed in [1], [2]). Asthma affects approximately 20 million people in the US and is now one of the most common chronic diseases of childhood, involving roughly 10% of children [3], [4]. While onset of asthma symptoms can be triggered by a myriad of initiating stimuli such as cold air, exercise, air pollutants and allergens, asthma exacerbations are now being increasingly linked to pulmonary infection with *Mycoplasma pneumoniae* (Mp) [5], [6], [7], [8].

Mp, the causative agent of “walking pneumonia,” often colonizes the airways of chronic asthmatics. In fact, recent studies report that greater than 50% of chronic stable asthmatics have evidence of airway infection with Mp [8] and that the majority of children taken to the emergency room during their initial asthmatic episode are colonized with Mp [9], [10]. While eosinophils have been implicated in asthma pathogenesis and eosinophil granular proteins are often found in bronchoalveolar lavage and serum in asthma patients, the contribution of eosinophils in Mp-induced exacerbations of allergic airways has not been previously described.

Surfactant protein-A (SP-A), a secretory product of airway and alveolar epithelial cells, is known to have important roles in host defense against microbes, although relatively little is known about the role of SP-A in asthma. Studies show that SP-A levels are reduced in asthmatics who are segmentally challenged with allergen [11] and previous studies from our lab show that SP-A deficient mice have enhanced airway hyperresponsiveness (AHR) and inflammation when infected with Mp alone [12]. Additionally, SP-A binds to Mp through phospholipids and through a specific binding protein [13], [14], further suggesting the importance of SP-A in mediating the immune response to this pathogen.

The goal of the current study is to evaluate the role of SP-A in mediating eosinophil responses to Mp infection in an allergic lung. Using the model antigen ovalbumin (Ova) to promote allergic airways followed by Mp infection (Ova+Mp), we find that eosinophils are potent killers of Mp and that SP-A benefits airway homeostasis by limiting eosinophil activation that may lead to lung inflammation and damage. However, SP-A also inhibits eosinophil-mediated clearance of Mp by reducing eosinophil peroxidase (EPO) release. Thus, we demonstrate for the first time that SP-A acts as a double-edged sword in the host response to Mp infection in the setting of allergic airway disease.

## Results

### Mp burden in Mp-infected allergic mice

**Figure 1:**
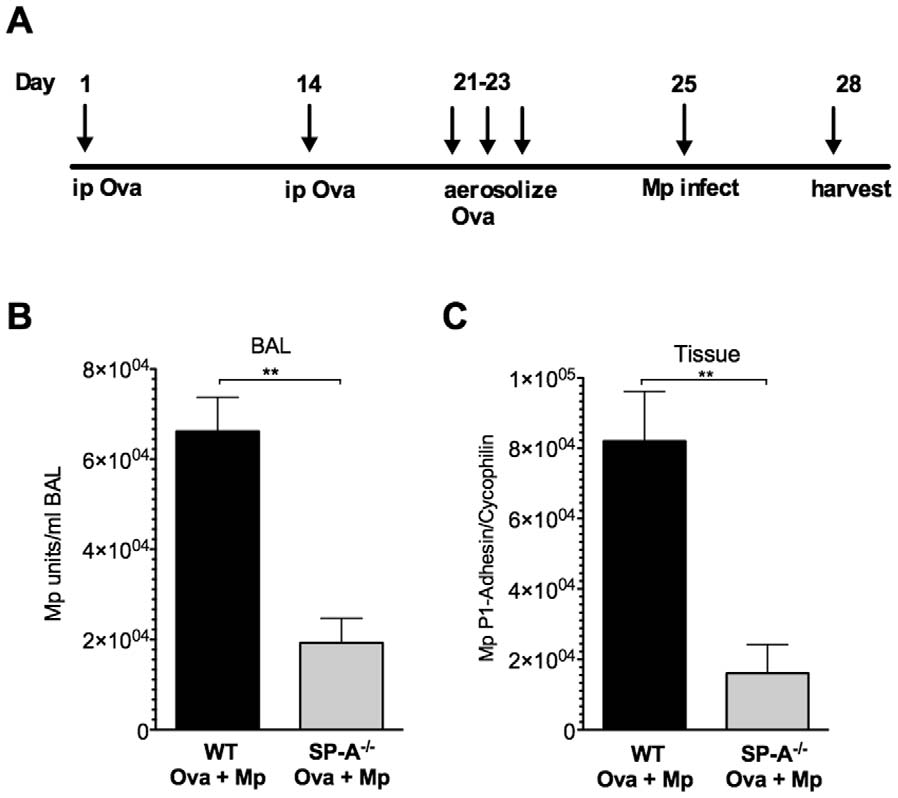
Mp burden is decreased in Mp-infected SP-A^**−/−**^ allergic mice. **A**) WT and SP-A^−/−^ mice were injected ip with Ova/Alum mixture on days 1 and 14, subject to Ova aerosolization on days 21–23 and instilled with either Mp or saline on day 25. Mp burden was determined in **B**) BAL by plating dilutions on PPLO agar plates and counting via 10× magnification or in **C**) lung tissue by RT-PCR for Mp P1-adhesin relative to housekeeper. n = at least 15 mice/group, 3 experiments combined, **p<.01.

**Figure 2:**
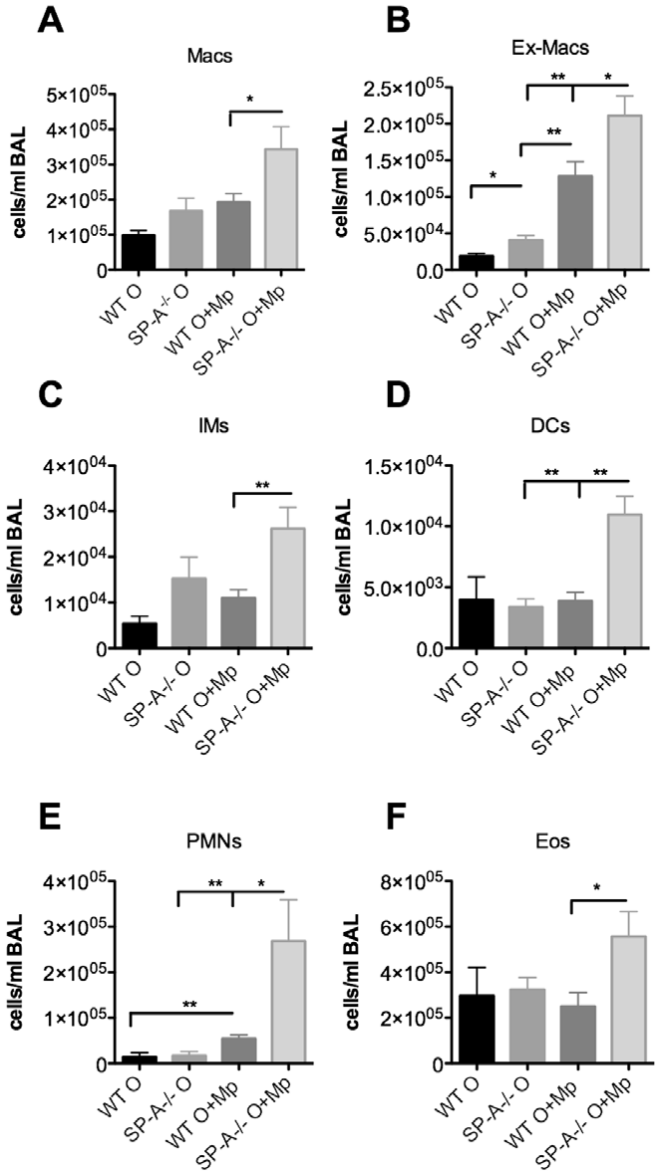
Recruitment of inflammatory cells is enhanced in Mp-infected SP-A^**−/−**^ allergic mice. **A–F**) Cells in the BAL were examined by cell surface labeling, as described in the methods section, and flow cytometry 3 days after Mp infection (day 28 of the model). Macs (macrophages), Ex-Macs (exudative macrophages), IMs (inflammatory monocytes), DCs (dendritic cells), PMNs (neutrophils), Eos (eosinophils). n = at least 12 mice/group, 3 experiments combined, *p<.05, **p<.01.

Previously, others have shown that by binding SP-A, Mp growth is restricted *in vitro* [13], [14], and our lab has shown that SP-A is vital in curtailing an overzealous TNF-mediated response to Mp by attenuating airway hyperresponsiveness and mucus production compared to WT infected mice [12]. To determine the role of SP-A in mediating the immune response to Mp in an allergic airway, we used the Ova-sensitization and challenge protocol, followed by intranasal infection with Mp (**fig. 1A**), similar to methods previously described [15]. Bronchoalveolar lavage (BAL) was plated to obtain CFUs and RT-PCR was performed from whole lung cDNA for Mp specific P1-adhesin gene. Surprisingly, SP-A^−/−^ mice had a significantly lower Mp burden measured in both BAL (**fig. 1B**) and lung tissue samples three days post infection (**fig. 1C**). This is in contrast to our previously published findings in non-allergic airways, such as those infected and harvested prior to the ova challenge on day 20 (no eosinophils are present in the BAL or tissue) where Mp burden is similar in BAL fluid in WT and SP-A^−/−^ mice (∼1×10^4^ Mp CFU/ml), and significantly greater in the airway tissue (∼20 fold greater) when SP-A is absent [12]. Our findings in allergic airways show that SP-A^−/−^ mice have decreased Mp burden as compared to WT mice in the BAL and lung tissue, as well as higher numbers of eosinophils three days post infection (**fig. 2F**). Mp infection was resolving by day seven in allergic WT and SP-A^−/−^ mice as Mp burden was similarly decreased in BAL and lung tissue compared to three days post infection in both groups of mice (data not shown). Taken together, these findings suggest that eosinophils, which are only found in allergic airways, are the key cell type leading to the decreased Mp burden in SP-A^−/−^ allergic mice and when more eosinophils are present, such as in the SP-A^−/−^ mice, less Mp remain. In non-allergic airways, where no eosinophils are present, more Mp burden persists in SP-A^−/−^ mice as compared to WT mice, likely due to the absence of SP-A to bind Mp and help resolve infection through mucociliary clearance mechanisms. Taken together, these findings also suggest that SP-A mediates Mp clearance by different mechanisms in non-allergic versus allergic lungs.

### Recruitment and activation of eosinophils

In order to determine which cell types could be responsible for the increased Mp killing/clearance in the allergic SP-A deficient mice, we compared cell populations from WT and SP-A null mice that were Ova sensitized/challenged and infected with Mp and to controls that were either exposed to the Ova challenge only (no Mp) or given saline. There were no significant differences between WT and SP-A^−/−^ mice in any of the cell populations examined in either saline treated or saline aerosolized mice (data not shown). Using flow cytometry of cells stained with antibodies against specific cell surface markers, we found that there were significantly more macrophages (Macs), CD11b+ exudative macrophages (Ex-Macs), inflammatory monocytes (IMs), dendritic cells (DCs), neutrophils (PMNs) and eosinophils (Eos) present in BAL of allergic/infected SP-A null mice as compared to WT mice (**fig. 2A–E**), further suggesting an important regulatory role of SP-A in this allergic/infectious model.

Interestingly, we found no eosinophils in our previous studies examining Mp infection in a non-allergic airway lacking SP-A, although we did see increases in the other cell populations. Samples from the Ova+Mp experimental model were collected on day 28 (5 days after the last Ova aerosol in Ova only and in Ova+Mp treated mice), the time at which airway physiology measurements were also conducted. Previous publications from our lab have established that on day 24 of the Ova only model, SP-A^−/−^ mice have even more pronounced eosinophilia than their WT controls [16]. We therefore verified in control groups of mice that the number of eosinophils present 48 hrs after the last Ova aerosol (on day 25 when mice are infected with Mp) remained significantly elevated in SP-A^−/−^ mice as compared to WT mice, as has been previously published. The finding that eosinophils are decreased in the Ova only groups by day 28 of our model, as compared to the previously published findings on day 24 [16], suggests that eosinophilia is resolving in the Ova only control groups of WT and SP-A^−/−^ mice.

**Figure 3:**
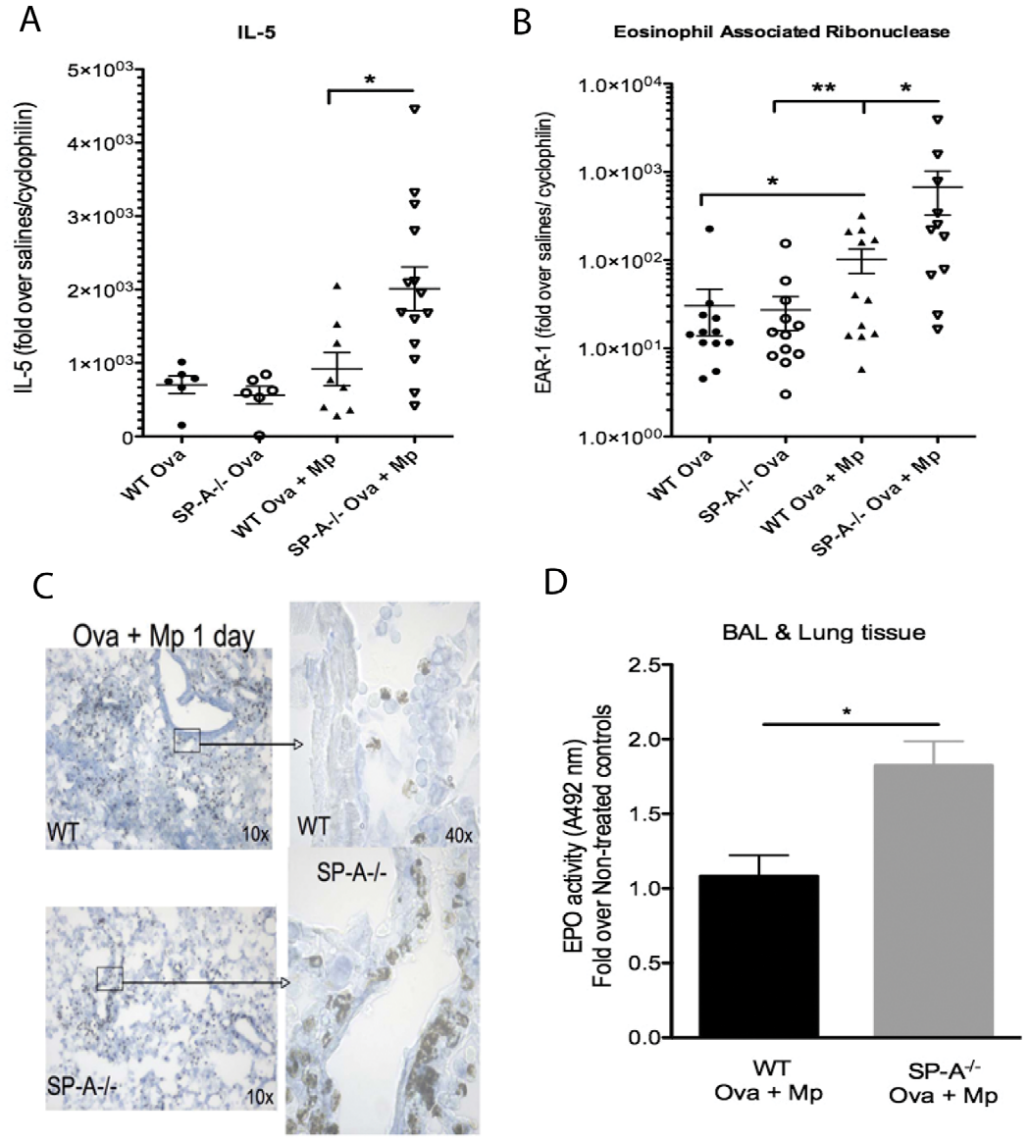
Eosinophil mediators are increased in the absence of SP-A. On day 28 of the Ova+Mp model, lungs were harvested and **A**) IL-5 and **B**) EAR were assessed by RT-PCR. **C**) Histochemical staining for EPO positive eosinophils was done 1 day after Mp infection and is representative of 3 experiments, n = 5/group. **D**) Total EPO activity in BAL and lung tissue from Ova+Mp mice was determined via colorimetric assay and absorbance read at 492 nm. n = combined 3 experiments, *p<.05, **p<.01.

Lungs were harvested from three groups of WT and SP-A null mice: 1) saline treated control mice, 2) Ova sensitized/challenged mice, 3) and Ova sensitized/challenged and Mp infected mice. To determine if SP-A regulates eosinophil activation to Mp infection in the allergic mice, markers of eosinophils, such as IL-5 and eosinophil associated ribonuclease (EAR), were analyzed by RT-PCR. In Ova allergic WT and SP-A^−/−^ mice, the levels of IL-5 and EAR RNA were dramatically but comparably increased over levels detected in samples taken from saline treated mice, suggesting similar numbers eosinophils were present in the lungs of WT and SP-A^−/−^ mice 5 days post Ova challenge. Interestingly, EAR expression, a marker typically associated with eosinophil activation, is significantly increased in the WT Ova+Mp compared to the WT Ova alone. This increase in EAR expression suggests that while the total eosinophil number is similar between the WT groups, the activation status of the eosinophils, as determined at the transcriptional level, in the WT Ova+Mp group is greater than in the WT Ova only. Additionally, when Ova allergic SP-A^−/−^ mice are infected with Mp, the levels of IL-5 and EAR RNA significantly increased over levels detected in the Ova allergic WT infected mice. This suggests that not only are more eosinophils present in the SP-A^−/−^ Ova+Mp mice, but also that SP-A may reduce the activation of eosinophils at the transcriptional level (**fig. 3A,B**).

Lung sections were analyzed from Ova sensitized/challenged Mp infected SP-A^−/−^ and WT mice by histochemical staining for cyanide resistant EPO activity. While the majority of EPO activity staining is observed in the lung parenchyma in the allergic infected WT mice (**fig. 3C**, upper panel), more staining was evident around the large airways of allergic infected SP-A^−/−^ mice, in close proximity to where we and others have shown Mp to colonize [12] (**fig. 3C**, lower panel). Additionally, EPO activity was measured in both BAL and lung tissue from Mp-infected allergic mice. The total EPO activity measured in the infected allergic mice lacking SP-A was significantly greater than that in the control WT mice given the same treatment (**fig. 3D**).

### Binding of SP-A to eosinophils

**Figure 4:**
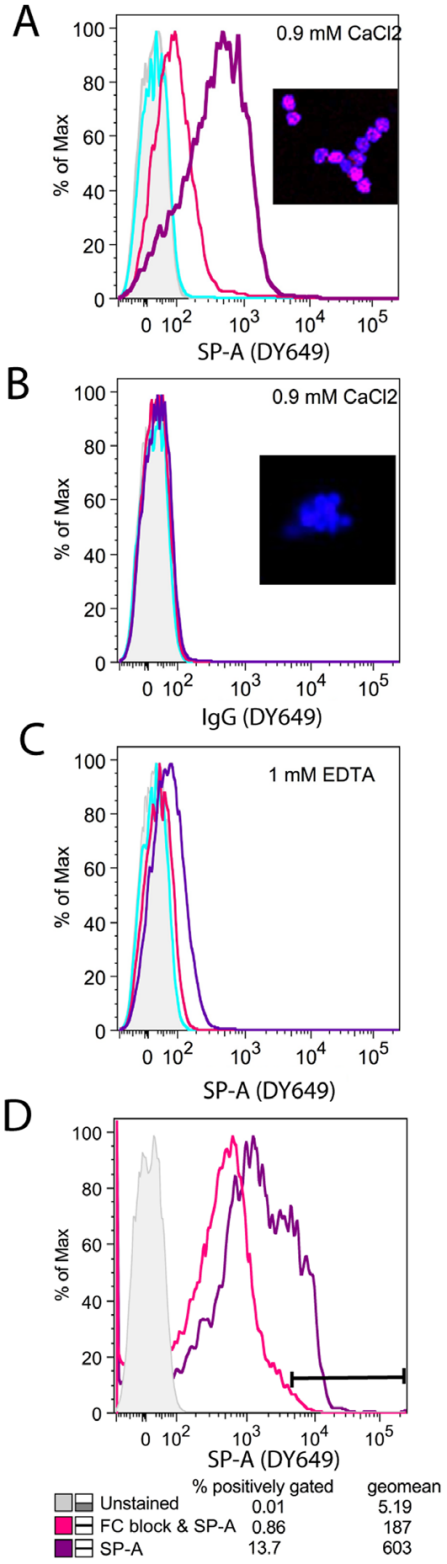
SP-A binds to eosinophils in a Calcium-dependent manner. Eosinophils were incubated with **A**) fluorescent labeled SP-A-DY649 (shaded = unstained cells, turquoise = 0.1 µg/ml, pink = 1 µg/ml, purple = 10 µg/ml) in calcium-rich buffer or **B**) fluorescent labeled IgG in calcium-rich buffer (at the same concentrations used above) or **C**) fluorescent labeled SP-A in calcium depleted buffer and binding was assessed by flow cytometry. SP-A binding (10 µg/ml) to labeled eosinophils was also observed with confocal microscopy as shown in the insets. DAPI (blue stain) and SP-A-DY649 (pink stain) stained slides were examined at 40× via confocal microscopy. **D**) SP-A binding of the percentage of highly positive cells (gated at 5×10^3^) was assayed by flow cytometry (geomean of DY649) after cells were pre-incubated with FC antibody (pink line) versus SP-A alone (purple line).

In order to assess the possibility that SP-A acts directly on eosinophils to modulate their responses, we sought to determine if SP-A binds to eosinophils. Eosinophils were purified from the blood of IL-5 transgenic mice and their purity determined to be greater than 95% by cytospin analysis of H&E staining and/or by positive cell surface labeling with the CCR3 antibody by flow cytometry. SP-A bound to eosinophils in a dose dependent manner (**fig. 4A**); only minimal binding of a control protein, non-immune IgG control was detected (**fig. 4B**). To test the Ca^2+^ dependency of SP-A binding to eosinophils, assays were performed in the presence or absence of EDTA. Binding of SP-A to eosinophils was optimal in Ca^2+^-rich buffer (**fig. 4A**) and was significantly repressed with the addition of EDTA (**fig. 4C**). Images visualized with a confocal microscope also demonstrate the abundance of SP-A binding to eosinophils versus that of the IgG control (**inset panels**). SP-D has been reported to bind eosinophils via the FC receptor [17]. To determine if SP-A also binds eosinophils through the FC receptor, binding of SP-A was determined after eosinophils had been pre-incubated with an FC-antibody. Binding of SP-A to eosinophils was dramatically reduced when cells were incubated with the FC antibody prior to the addition of SP-A as shown by flow cytometry (**fig. 4D**).

### Involvement of eosinophils in Mp killing

In contrast to our previously published data showing that SP-A^−/−^ mice had significantly more Mp colonizing the large airway compared to WT mice, the SP-A^−/−^ mice challenged with Ova and infected with Mp have significantly decreased Mp burden compared to WT Ova+Mp challenged mice. Interestingly, eosinophils are only present in the infected allergic mice (not present in the Mp infected only) and are significantly higher in the BAL of SP-A^−/−^ mice compared to WT mice at the time of Mp instillation and at the time of harvest 3 days post infection. These findings suggest that eosinophils may be important mediators of Mp clearance in allergic airways.

**Figure 5:**
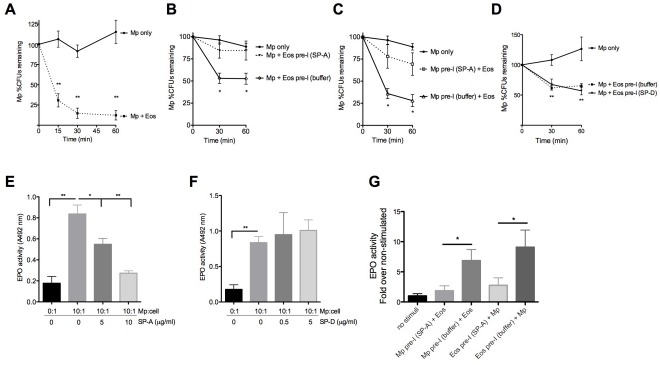
Eosinophil-mediated Mp killing is attenuated by exogenous SP-A. **A**) Purified eosinophils were added to Mp (10∶1) for 1 hr and aliquots were diluted on PPLO agar plates for CFU counts. **B**) Mp and **C**) Eosinophils were pre-incubated with SP-A (10 µg/ml) for 30 minutes to allow for binding and centrifuged prior to their addition to the killing assay. **D–F**) SP-D (0.5–5 µg/ml) or SP-A (5–10 µg/ml) was pre-incubated with eosinophils prior to the addition of Mp MOI 10∶1. EPO was measured in the supernatant after 1 hr of stimulation. **G**) Mp or Eosinophils were pre-incubated with SP-A (10 µg/ml) for 30 minutes to allow for binding and centrifuged prior to their addition to the EPO stimulation assay *p<.05, **p<.01, n = 3 experiments.

In order to determine if eosinophils kill Mp, eosinophils were purified from the blood of IL-5 transgenic mice and co-incubated with Mp for 1 hour. In the first 15 minutes, ∼75% of the Mp was killed by eosinophil-mediated mechanisms, and by 60 minutes less than 10% of Mp remained viable (**fig. 5A**). Additionally, pre-incubation of eosinophils with a physiological concentration of SP-A prior to Mp addition, interfered with their killing mechanisms as compared to control eosinophils incubated with control buffer (**fig. 5B**). These experiments support the *in vivo* data where we observed less Mp burden when more eosinophils are present (as in the SP-A^−/−^ mice). Since SP-A is known to bind Mp, we did the reverse experiment in which SP-A was pre-incubated with Mp prior to addition to eosinophils in culture. SP-A bound to Mp also decreased the ability of eosinophils to kill Mp optimally when compared to the Mp that was pre-incubated with the buffer control (**fig. 5C**). Since both sets of experiments, eosinophils pre-incubated with SP-A and Mp pre-incubated with SP-A, were centrifuged after the pre-incubation to remove any unbound SP-A, our findings suggest the binding of SP-A to either eosinophils or Mp is sufficient to interfere with eosinophil-mediated Mp killing. Pre-incubation with SP-D did not affect the ability of eosinophils to kill Mp (**fig. 5D**).

### SP-A inhibits EPO release from Mp-stimulated eosinophils

EPO has long been known to be an important agent against multicellular parasites and some bacteria, including *M. tuberculosis* [18]. Significantly more EPO activity was found in samples harvested from infected allergic mice lacking SP-A as compared to WT mice with the same treatment. Since more eosinophils in the SP-A^−/−^ infected allergic mice could account for the increased EPO, we did *in vitro* studies to determine if exogenously added human SP-A inhibits EPO release from activated eosinophils. While stimulation of eosinophils with Mp resulted in significant EPO activity in the supernatant, SP-A pre-incubation at physiologic concentrations with the eosinophils reduced the amount of EPO released dose-dependently (**fig. 5E**). The viability of the cells was assessed and there were no significant differences in any of the treatment groups (all groups had less than 5% cytotoxicity) indicating that cell death was not a cause of increased EPO release (not shown). As a control, another surfactant known to bind eosinophils, SP-D [17], used at physiologic concentrations, was unable to inhibit EPO release from Mp-stimulated eosinophils (**fig. 5F**) suggesting that the inhibition of Mp-stimulated EPO release is not a shared function of the lung collections. As described for eosinophil mediated Mp killing above, binding of SP-A to either Mp or to the eosinophils prior to their addition to the assay resulted in decreased EPO release into the supernatant (**fig. 5G**), supporting the importance of direct interaction of Mp with the eosinophil to initiate EPO release.

### Levels of surfactant proteins during allergy and infection

**Figure 6:**
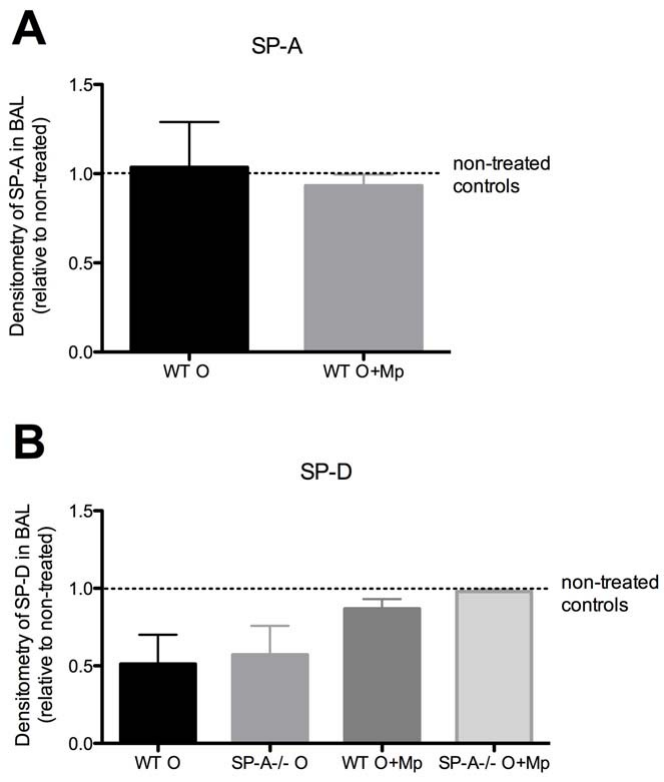
Alteration of surfactant levels during inflammation and infection. **A**) SP-A or **B**) SP-D levels were determined by densitometry of Western blots of BAL samples of untreated, Ova only (day 28 of our model) or Ova+Mp (day 28 of our model). n = 2 experiments with 3–4 mice/group each.

Surfactant levels are known to change in certain inflammatory conditions. Since alterations in either SP-A or SP-D levels during allergic and infectious conditions could potentially influence the killing capacity of eosinophils, we sought to determine levels of each surfactant in the BAL of allergic and allergic/infected mice using densitometry measurements of Western blots. SP-A levels were similar in WT mice among untreated controls, Ova treated (day 28 of our model: 5 days after the last aerosol) and in Ova+Mp treated (day 28 of our model: 5 days after the last aerosol/3 days after Mp infection) (**fig. 6A**). SP-D levels were decreased similarly (although they did not achieve statistical significance) in both WT and SP-A^−/−^ mice in Ova treated mice as compared to untreated controls (**fig. 6B**). However, in Ova+Mp treated mice, levels of SP-D were similar to untreated controls (**fig. 6B**). These findings show that five days after the last Ova aerosol in non-infected and in Mp-infected mice, SP-A levels remain similar to levels in untreated mice. Additionally, SP-D levels are not decreased during Ova+Mp conditions as compared to untreated controls and the amount of SP-D is not significantly different between WT and SP-A^−/−^ mice in any of the treatment groups examined, suggesting a limited contribution of SP-D to the observed killing phenotype.

### Contribution of EPO to Mp killing

**Figure 7:**
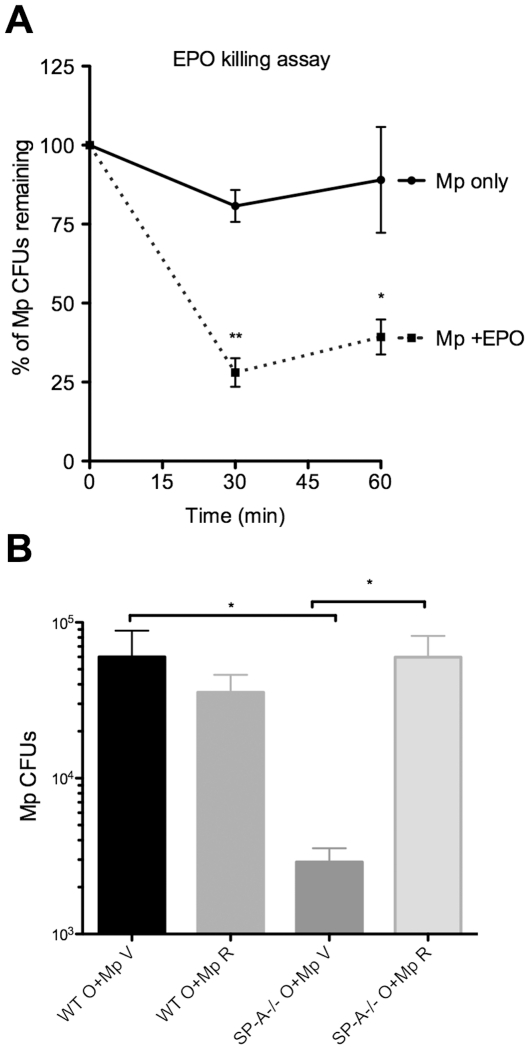
EPO is vital for Mp killing mechanisms *in vitro* and *in vivo*. **A**) Purified human EPO (0.5 µM) was added to Mp (5×10^6^) and viability was assessed over the course of 1 hr. **p<.01,*p<.05. n = 3 experiments. **B**) Mice were treated with Ova and Mp (O+Mp) as previously described but 2 hrs prior to Mp infection, some mice were given either vehicle (V) or resorcinol (R). Mice were given boosters after 24 hrs and samples harvested at 72 hrs for Mp burden. *p<.05. n = 2 experiments (8–10 mice/group).

Our *in vivo* data suggests that the presence of SP-A appears to interfere with eosinophil mediated killing of Mp and our *in vitro* data suggests that one mechanism through which SP-A exerts this affect is by binding eosinophils and limiting EPO release when they encounter Mp. Therefore, to determine if EPO can directly kill Mp, purified EPO was added to Mp cultures for 1 hour and Mp viability was assessed. When used at a similar concentration (0.5 µM) reported to kill *M. tuberculosis* [18], EPO killing of Mp appears to be rapid, with 75% of the pathogen eliminated in the first 30 minutes of incubation (**fig. 7A**).

Because we observed EPO kills Mp *in vitro* and because SP-A^−/−^ mice have more EPO present in BAL and lung samples and less Mp burden, we tested whether an inhibitor of EPO activity would result in decreased clearance of Mp in the allergic SP-A^−/−^ mice. Mice were sensitized and challenged with Ova as described above. Immediately prior to Mp infection, mice were given an injection of the peroxidase inhibitor, resorcinol, as previously described [19]. Resorcinol treatment did not affect the percentage of eosinophils recruited in the BALs (data not shown). As expected, Mp infected allergic SP-A^−/−^ mice given vehicle (PBS) had significantly less Mp burden than Mp infected allergic WT mice given vehicle (**fig. 7B**). However, allergic SP-A^−/−^ mice given resorcinol were unable to clear the Mp as well as allergic SP-A^−/−^ mice given vehicle (**fig. 7B**) suggesting that the increased clearance observed in the SP-A^−/−^ mice could be attributed to increased EPO activity.

### Mucus production and lung damage in Mp-infected allergic airways

**Figure 8:**
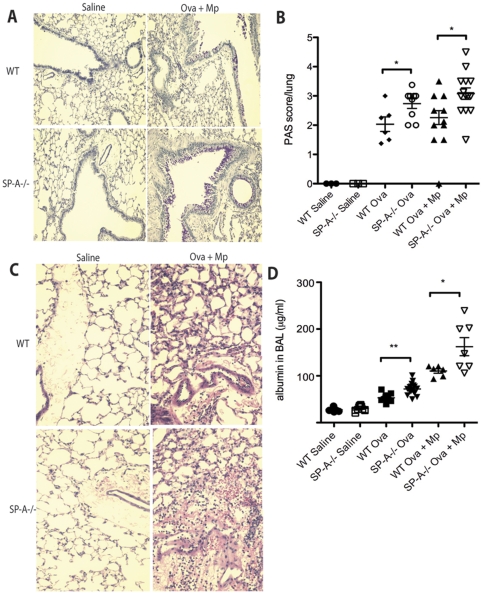
SP-A is protective against Mp-induced allergic pathologies. **A**) PAS stained lung sections examined at 10× magnification were **B**) blindly scored for mucus production with 0 representing no mucus present and 5 being mucus present in greater than 75% of airways and bronchioles. **C**) H&E stained lung sections shows inflammation and RBCs in alveolar spaces and lymphatics of Ova+Mp treated mice. **D**) Albumin measured in the BAL as a measure of lung damage and vascular permeability. n = representative of 3 experiments, *p<.05.

A part of the immune response to Mp infection in an allergic airway is dramatically heightened mucus production by goblet cells. While PAS stained cells were increased in Ova treated and in Mp-infected Ova treated airways of both WT and SP-A^−/−^ mice as compared to saline controls, SP-A^−/−^ mice had significantly more PAS positive cells evident even in large airways and distal bronchioles compared to WT mice in each of the treatment groups (**fig. 8A,B**). Resorcinol treatment did not affect the percentage of PAS positive cells in the large airways (data not shown). In conjunction with our previous data presented in this manuscript, this indicates there is no correlation between Mp burden and mucus production in the large airway in our mouse model.

Albumin levels in the BAL were measured as another gauge of lung damage and permeability. Albumin is one of the most abundant proteins in the bloodstream and as lungs are damaged, as in the case of severe inflammation, weakened pulmonary blood vessels allow leakage from the bloodstream into the lung tissue and airspaces. Typically, a heightened amount of albumin in BAL is thought to correlate with heightened levels of tissue damage and/or vascular permeability. Ova treated WT and SP-A^−/−^ mice had significantly increased albumin in BAL samples as compared to saline treated mice. Additionally, Ova treated mice lacking SP-A, had significantly more albumin in BAL as compared to WT Ova treated mice (**fig. 8D**). Not surprisingly, Mp-infected allergic mice either sufficient or deficient in SP-A had increased inflammation and albumin in their BAL compared to saline treated mice and to Ova treated mice (**fig. 8C,D**). However, mice lacking SP-A had more pronounced red blood cells infiltrating the lung parenchyma (**fig. 8C**) and significantly greater levels of BAL albumin than did WT mice (**fig. 8D**).

## Discussion

Mp is currently believed to be one of the most common pathogens linked to asthma exacerbations (reviewed in [20]). Studies in mice have demonstrated that infection of an Ova allergic airway with Mp augments airway hyperreactivity and inflammation compared to that observed in an Ova allergic airway alone [15], providing additional evidence for the link between Mp infection and chronic asthma as observed in humans. The present work shows that SP-A plays a dual role in allergic airways with Mp superinfection. SP-A protects the airway by limiting cellular inflammation, eosinophil activation and release of eosinophil products such as EPO, all of which may lead to airway damage. However, perhaps as a consequence of this anti-inflammatory role, SP-A simultaneously appears to interfere with eosinophil-mediated biologic clearance of Mp by curtailing EPO-driven killing mechanisms.

The release of cationic proteins from activated airway eosinophils has been implicated as a possible mechanism contributing to exacerbations in chronic asthmatics. Granular products, such as MBP and EPO, are thought to induce epithelial damage and airway constriction, and EPO itself has been linked to airway remodeling [21]. However, it is unknown if Mp colonization of eosinophil-laden airways, as is common in many asthmatics, results in eosinophil activation and subsequent degranulation. Prior publications have reported that eosinophil degranulation does not occur in the Ova challenge model in mice [22], which our data support. However, when Mp infects an Ova challenged airway, we find evidence of eosinophil activation, as shown by increased EAR transcription, and increased EPO in BAL and tissue samples, suggesting that eosinophil degranulation has also occurred.

Surfactant proteins have long been recognized as modulators of the innate immune system in part by directly binding to a variety of inflammatory cell types [23], [24], [25], [26]. Discrepancies exist in the field regarding the levels of surfactant proteins after allergen provocation. Surfactant proteins measured in BAL from humans with asthma have been reported to both increase and decrease (reviewed in [27], [28]). Recent studies, however, have shown that in segmentally challenged asthmatics, SP-A levels are decreased while SP-D levels were increased [11]. Previous work by Schmiedl *et al* showed increases in both SP-A and SP-D acutely in an Ova model [29]. However, in our model of Ova challenge when we examine surfactant levels during the resolution phase (5 days post challenge), we find that SP-A levels are similar to untreated controls while SP-D levels are slightly decreased.

Current studies in the field suggest that SP-A may be dysfunctional in several lung diseases such as Respiratory Distress Syndrome, Idiopathic Pulmonary Fibrosis, and asthma possibly due to genetic variants [30], [31], [32], [33], [34], [35]. This decrease in functional SP-A in asthmatics may be associated with increases in Mp-induced exacerbations seen in allergic airways as compared to Mp infections in non-allergic airways. Additionally, Mp clearance in non-allergic airways is typically driven by macrophages and mucocilliary actions while eosinophils are rarely present [36]. In contrast, eosinophils are commonly present in the airways of asthmatics where they will encounter invading Mp. Based on our findings in mice, if SP-A is absent or dysfunctional in the asthmatic airway, colonization with Mp would most likely result in eosinophil activation and degranulation. We are the first to report that SP-A binds eosinophils directly and inhibits their release of EPO. While the limited release of EPO may interfere with the body's natural response to clear Mp, we speculate that decreased release of other harmful cationic products will aid in protecting the airway from damage due to excessive inflammation.

Previous work by our lab showed that SP-A regulated the interaction between Mp and DCs via TLR-2, and SP-A pre-incubated and bound to Mp was critical in limiting Mp from interacting with TLR-2 on dendritic cells [37]. Interestingly, pre-incubating dendritic cells with SP-A was not as effective at reducing Mp stimulation. In the case of eosinophils, however, SP-A binding to either the eosinophil or to the Mp seems to be important in regulating the EPO-driven killing activities of the eosinophils. This suggests the engagement of Mp with the eosinophil is critical in EPO release and subsequent killing of Mp by the eosinophil. When this engagement is altered with either SP-A binding to the eosinophil or with SP-A binding to the Mp, EPO release and killing of Mp by the eosinophil is limited.

Recent findings show that intestinal eosinophils express high levels of SIRP-α, an inhibitory receptor signal regulatory protein, and that cross-linking of SIRP-α on the surface of eosinophils significantly reduced the amount of EPO released during stimulation with a calcium ionophore [38]. While our findings demonstrate that SP-A binds eosinophils through the FC receptor, interestingly, SP-A has also been shown to bind directly to SIRP-α on the surface of other cells, such as macrophages [39]. Additionally, since Mp stimulates cells almost exclusively through TLR-2 [37], [40], SP-A may bind Mp and limit the ability of the Mp to signal via TLR-2, which results in decrease EPO release from the eosinophils. Future studies should investigate whether lung eosinophils express SIRP-α and whether the mechanism by which SP-A limits EPO release from Mp-stimulated eosinophils involves any of these receptors in which SP-A is known to interact.

While multiple cell types were increased in the SP-A^−/−^ Mp infected allergic mice compared to WT of the same treatment, we had strong reason to believe that eosinophils were responsible for the decreased Mp burden. First, in non-allergic SP-A^−/−^ mice infected with Mp the burden and colonization in the large airway was significantly greater than in WT mice [12]. In the Mp-infected (non-allergic) mice, many of the cell types in the SP-A^−/−^ mice were also significantly increased as compared to WT mice of the same treatment (macrophages, exudative macrophages, and dendritic cells are all increased in the SP-A^−/−^ mice; neutrophils and inflammatory monocytes are similar between WT and SP-A^−/−^ mice; no eosinophils are present). Although greater numbers of other inflammatory cells persist when SP-A is absent that could kill Mp, Mp burden in the lung tissue is significantly greater in SP-A^−/−^ mice [12]. This suggests that the same populations of cells present in the non-allergic lungs (macrophages, exudative macrophages, dendritic cells, neutrophils and inflammatory monocytes) are likely not contributing to the increased killing mechanisms we observe in the allergic lung. Thus, the only population different that we observe between the non-allergic and allergic lung cell populations that can kill Mp, are the eosinophils. Eosinophils numbers were increased only in the Mp-infected allergic model but not in the Mp-infected non-allergic model. While eosinophils were significantly increased at the time of harvest, 3 days post infection, sampling was done immediately prior to Mp infection in a group of mice and eosinophils present from the Ova challenge alone were even higher and significantly elevated in the SP-A^−/−^ mice as compared to WT controls. This is in agreement with previously published reports from our lab in the Ova sensitization and challenge model in SP-A^−/−^ versus WT mice [16].

In conclusion, our work demonstrates that Mp causes eosinophil activation and EPO release and that SP-A plays dual roles as both protective, by limiting these harmful responses, and intrusive, by inhibiting eosinophil mediated Mp killing. Mp infected mice lacking SP-A have increased inflammation, vascular permeability, and mucus production as compared to Mp infected mice sufficient in SP-A. While SP-A interferes with the ability of the eosinophil to naturally kill Mp by inhibiting the engagement of the eosinophil with Mp and thereby reducing EPO release, SP-A simultaneously protects the lung by limiting Mp-induced eosinophil activation and release of other potentially harmful products into the airway. Additionally, we are the first to show that eosinophils kill Mp through EPO-driven mechanisms and that when EPO is neutralized *in vivo*, Mp clearance is impaired. Thus, SP-A is pivotal in maintaining homeostasis in the pulmonary environment by preserving a fine balance between mounting host defense mechanisms while limiting an overzealous response that could ultimately prove damaging to the host.

## Methods

### Ethics Statement

All mouse studies were carried out in strict accordance with the recommendations in the Guide for the Care and Use of Laboratory Animals of the National Institutes of Health. The protocol was approved by the Institute of Animal Care and Use Committee (IACUC) at Duke University (protocol number A284-09-09, approved on 9/23/10). All surgery was performed under Ketamine (100 mg/kg)/Xylazine (5 mg/kg) anesthesia, and all efforts were made to minimize suffering.

### *M. pneumoniae* culture

Mp from ATCC (cat. #: 15531) was grown in SP4 broth (Remel) at 35°C (without CO_2_) and passaged until adherent. Mp concentration was determined by plating serial dilutions of Mp on PPLO plates (Remel). CFUs were counted under 10× magnification on plates after incubation for 7 to 14 days. For *in vivo* infection, adherent Mp was washed by centrifuging at 6000 rpm for 5 minutes and resuspended in sterile saline for infection. To determine Mp counts from the BALs, 10 µl were plated on PPLO plates and counts were recorded as described above after 14 days of incubation.

### RT-PCR

Lung tissue was collected and RNA extracted by phenol-chloroform methods. cDNA was prepared according to standard protocols and the amount of Mp was detected by RT-PCR by using primers specific for the Mycoplasma pneumonia specific P1-adhesion gene (forward 5′ CGC CGC AAA GAT GAA TGA C 3′, reverse 5′ TGT CCT TCC CCA TCT AAC AGT TC 3′). Primers for IL-5 (forward 5′ AGC ACA GTG GTG AAA GAG ACC TT 3′, reverse 5′ TCC AAT GCA TAG CTG GTG ATT T 3′) and EAR (forward 5′ CGA CTT TGT CTC CTG CTG 3′, reverse 5′ TGT CCC ATC CAA GTG AAC 3′) were used. Relative amounts of Mp P1-ahdesin, IL-5 or EAR present in each infected lung were measured based on values obtained from a standard curve for Mp P1-adhesin and were normalized to the mammalian housekeeping gene cyclophilin or to ß-actin that was present in the lung tissue.

### Mice

Wild type and SP-A^−/−^ mice were maintained on a C57Bl/6 (Charles River) background and were 8–12 weeks of age at the time of harvest. SP-A^−/−^ mice were generated by homologous recombination as described previously [41]. IL-5 transgenic mice (NJ1638) were a generous gift from Dr. J.J. Lee, Mayo Clinic [42]. All mice used in experiments were on protocols approved by the Institutional Animal Care and Use Committee at Duke University.

### Ova+Mp infection model

WT and SP-A^−/−^ mice were sensitized via intraperitoneal (i.p.) injections of Ova (Sigma) emulsified in Aluminum Hydroxide gel (Sigma) on days 1 and 14 and challenged with Ova aerosol on days 21–23 (1% aerosol nebulizer). Mice received either saline or Mp instillation (1×10^8^ in 50 µl sterile saline under anesthesia via ketamine/xylazine mix) on day 25, similar to published protocols [15], [43], to evaluate role of SP-A in Mp infection during allergic airway disease. Some mice received i.p. injections of resorcinol (1.25 mg/kg) or vehicle (PBS) 2 hours prior to Mp infection and 24 hours following infection [19].

### Isolation of BALs and FACs Analysis

Mice were euthanized with a lethal dose of Nembutal. To quantify cells in lung of uninfected SP-A^−/−^ and WT mice, animals were subjected to bronchoalveolar lavages with 6 mls of 37°C PBS (with 0.2 mM EDTA). BALs were resuspended in RBC lysis buffer (150 mM NH_4_Cl, 10 mM KHCO_3_, 0.1 mM EDTA) and incubated on ice for 10 minutes. Cells were then resuspended in buffer (HBSS −Ca/−Mg, 10 mM EDTA, 5% FCS) and counted using a hemacytometer. Flow cytometry was performed using a BD LSRII at the Duke Human Vaccine Institute Flow Cytometry Core Facility that is supported by the National Institutes of Health award AI-51445. BAL cells were incubated with fluorescent antibodies against specific cell surface markers: FITC-MHC II, PE-Ly-6G, and APC-Cy7-GR-1 (BD Biosciences), PE-Cy5-CD11c and APC-CD11b (eBioscience), PE-CCR3 (R & D Systems). Macrophages were identified based on FSC vs SSC, FITC autofluorescent, MHC II^lo-med^, CD11c^+^; Ex-Macs were identified the same as macrophages but that are also CD11b+; IMs were identified as Ly-6G^−^, MHC II^−^, CD11c^−^, GR-1^+^, CD11b^hi^; DCs were identified as MHC II^hi^, CD11c^hi^; PMNs were identified as Ly-6G^+^, GR-1^+^; Eos were identified as CCR3^+^, CD11b^+^.

### Determination of Albumin in Mouse BAL Samples

Mouse albumin concentrations were determined using an immunoperoxidase assay from Immunology Consultants Laboratory, Inc. Briefly, BAL samples were diluted 1∶100,000 and incubated on a micro-titer plate that had rabbit anti-mouse albumin polyclonal antibody bound to solid phase while detection was based on streptavidin-peroxidase polymer. The plate was read at 450 nm. The background value was subtracted from the test values for each sample and the test sample values were interpolated from the standard curve and multiplied for the dilution factor.

### Histological Analysis

On day three after Mp infection, WT and SP-A^−/−^ mice were euthanized by a lethal dose of Nembutal followed by exsanguination. The lungs were then perfused with 10 mls of warm PBS and inflated by gravity flotation with 4% paraformaldehyde fixative. Lungs were paraffin embedded, cut at 8 µm, and stained with either H&E or PAS. Histochemical staining for eosinophils was done on OCT frozen sections, cut at 10 µm as previously described [44]. Briefly, sections were incubated for 8 minutes at room temperature in PBS supplemented with 3,3-diaminobenzidine tetrahydrochloride (60 mg/100 ml; Sigma), 30% H_2_O_2_ and NaCN (120 mg/100 ml; Sigma). Slides were immersed in water, counterstained with hematoxylin, mounted, and eosinophils identified based on their dark brown reaction product. Slides that were assessed for PAS stain were done so in a blinded manner and rated on a scale of 0–5. A score of 0 had no visible PAS stained cells, as seen in the saline groups. Images of the lung were taken on a Nikon Eclipse 50i light microscope at ×10 (aperture 0.3) or ×40 (aperture 0.75) at room temperature by digital photography of bright-field images using a Nikon Infinity 2 camera. Infinity Capture software was used for image acquisition, and Adobe Photoshop was used for color-contrast settings and figure presentation.

### Eosinophil purification

Briefly, blood was collected via cardiac puncture from an IL-5 transgenic mouse into 0.5 ml EDTA tubes (Sarstedt). RBC were lysed and cells were resuspended in 0.1% BSA in PBS. Biotin labeled antibodies (1 µl per 10^7^ cells) against B220 (CD45R) and Thy 1.2 (CD90.2) were incubated with cells for 15 minutes on ice. Cells were then resuspended in 0.1% BSA and Dynabeads were added (1 µg/10^6^ target cells up to 50 µl) and incubated for 30 minutes at 4°C with tube rotation every 5 minutes. Samples were then placed into the magnetic apparatus with 4 mls additional media for 5 minute increments to deplete the magnetically labeled cells, leaving a greater than 95% pure population of eosinophils.

### EPO activity assay

Purified eosinophils (∼5×10^5^ per well) were stimulated with Mp (5×10^6^ per well) for 1 hour in the presence or absence of different concentrations of SP-A or SP-D. EPO activity was measured based on methods that were adapted from previously published protocols [22]. Briefly, 25 µl of either BAL, tissue homogenate, or cell supernatant was added to a reaction buffer (50 mM Tris-HCl, 4 mM H_2_O_2_, 0.1% Triton X, 10 mM O-phenylenediamide) in the presence or absence of the peroxidase specific inhibitor, 3-amino-1,2,4-triazole (30 mM). The reaction was allowed to proceed for 15–30 minutes at 37°C after which time stop solution (2N H_2_SO_4_) was added and the plate read at 490 nm.

### Eosinophil killing assay

Methods were adapted from Persson *et al* [45]. Briefly, experiments were done in a 96-well plate setup and with ∼5×10^6^ Mp were used per sample well. Some wells received buffer controls while other received purified eosinophils at a concentration of approximately 1 eosinophil per 10 Mp (∼5×10^5^ cells: 5×10^6^ Mp). Some eosinophils were pre-incubated with SP-A at a concentration of 40 µg/ml for 30 minutes and centrifuged at 1200 rpm for 5 minutes prior to their addition to Mp. Other eosinophils (not incubated with SP-A) were also incubated for 30 minutes and centrifuged at 1200 rpm for 5 minutes in order to maintain the same conditions throughout the treatment groups. Viability of eosinophils was assessed at the end of each assay by LDH (Abcam) testing in the supernatant. All samples had low and equal amounts of LDH released during the hour-long incubation. Some experiments were carried out with purified EPO (Lee Biosolutions, Inc.) at a concentration of 0.5 µM, similar to previously published methods [18].

### SP-A preparation

SP-A was purified from the lung lavage fluid of patients with alveolar proteinosis as described previously [46]. Briefly, the lavage fluid was initially treated with butanol to extract the SP-A. The resulting pellet was then sequentially solubilized in the detergent octylglucoside and 5 mM Tris, pH 7.4. Extracted SP-A was then passed over a polymyxin B-agarose column to reduce endotoxin contamination. SP-A preparations had final endotoxin concentrations of <0.01 pg/mg SP-A as determined by the Limulus amoebocyte lysate assay according to manufacturers' instructions (QCL-1000, BioWhittaker (Lonza)). Some SP-A was fluorescently labeled with DyLight Dy649 N-hydroxysuccinimide ester (Pierce Thermo Fisher) in a Slidealyzer G2 10000 MWCO cassette at pH 6.36 to maintain biological function. Excess reactive dye was dialyzed out in PBS, pH 7.4 with three changes of 500-fold excess buffer. The density of labeling averaged 2.6, using an extinction coefficient of 72000 for SP-A.

### SP-A binding to eosinophils

Once eosinophils were purified, approximately 5×10^5^ eosinophils were placed into tubes in either Ca^2+^ rich (0.5% BSA, 0.9 mM CaCl2) or Ca^2+^ depleted (0.5% BSA, 1 mM EDTA) media. Different concentrations of either fluorescent SP-A or IgG were added ranging from 0.1–10.0 µg/ml. Samples were incubated on ice for a minimum of 30 minutes prior to centrifugation at 1200 rpm for 5 minutes. Cells were then fixed in 2% formalin and examined by flow cytometry. All binding studies were done on ice to prevent eosinophil internalization of SP-A or IgG.

### Statistical Analysis

All data measurements were analyzed with PRISM software (GraphPad), first to determine if data were normally distributed, followed by t-test to determine significance. Data sets with significant variance between groups were analyzed by t-test using Welch's correction as assessed in PRISM. Statistical values of *p<.05 and **<.01 unless otherwise noted.
